# Analysis of Differentially Expressed Genes in Tissues of *Camellia sinensis* during Dedifferentiation and Root Redifferentiation

**DOI:** 10.1038/s41598-019-39264-5

**Published:** 2019-02-27

**Authors:** Ying Gao, Min Zhao, Xiao-Han Wu, Da Li, Devajit Borthakur, Jian-Hui Ye, Xin-Qiang Zheng, Jian-Liang Lu

**Affiliations:** 1 Zhejiang University Tea Research Institute, Hangzhou, 310058 P.R. China; 2 The World Vegetable Centre, Guwahati, Assam India

## Abstract

Tissue culture is very important for identifying the gene function of *Camellia sinensis* (L.) and exploiting novel germplasm through transgenic technology. Regeneration system of tea plant has been explored but not been well established since the molecular mechanism of tea plant regeneration is not clear yet. In this study, transcriptomic analysis was performed in the initial explants of tea plant and their dedifferentiated and redifferentiated tissues. A total of 93,607 unigenes were obtained through *de novo* assembly, and 7,193 differentially expressed genes (DEGs) were screened out from the 42,417 annotated unigenes. Much more DEGs were observed during phase transition rather than at growth stages of callus. Our KOG and KEGG analysis, and qPCR results confirmed that phase transition of tea plant was closely related to the mechanism that regulate expression of genes encoding the auxin- and cytokinin-responsive proteins, transcription factor MYB15 and ethylene-responsive transcription factor ERF RAP2-12. These findings provide a reliable foundation for elucidating the mechanism of the phase transition and may help to optimize the regeneration system by regulating the gene expression pattern.

## Introduction

Tea plant (*Camellia sinensis* (L.) O. Kuntzes) is one of the most important woody crops worldwide and getting popular because of the health benefit effect of the non-alcoholic beverage made from its tender shoot. It is very difficult to elucidate the genetic bases of the main economic traits and shorten the time for cultivar improvement since self-incompatibility and long cycle of seed to seed of the tea plant. Transgenetic technology is a useful tool to understand genetic mechanisms of the traits and accelerate innovation of the germplasms. Unfortunately, there are many obstacles in application of transgenic technologies in understanding development mechanism of the tea plant such as low transformation frequency and absence of a robust plant regeneration system. Calli, buds and embryoids have been successfully induced from the cotyledons^1^, cotyledon petioles, hypocotyls, immature embryos, axillary buds and immature leaves^2^, as well as, from stems and anthers^3^. Although plantlets had also been regenerated through organogenesis and somatic embryogenesis, a significant difference in regeneration frequency was observed from various explants^4^, and very low frequency was usually witnessed during induction of many explants. Studies on mechanism of dedifferentiation and redifferentiation during tissue culture may help to optimize high-frequency regeneration system of the tea plant.

Phase transition, a term widely used in plant development and cell cycle, has also been extendedly used in tissue culture and micropropagation to express the change among the stage of explants, dedifferentiated and redifferentiated tissues^5–8^. Phase transition during tissue culture is remarkably influenced by many factors, especially by plant hormones. Auxins and cytokinins (CKs), as important plant hormones, play crucial roles in DNA duplication, metabolism of nucleic acids, synthesis of various proteins, as well as mitosis and cytokinesis^9,10^. In addition to being considered as the essential elements for plant tissue culture, auxins and CKs have been used for the induction of calli and plantlet regeneration. Many studies in model plants revealed that hormones influence several levels of regulation, such as signal transduction and gene expression regulation. During root induction of *Arabidopsis* by auxin, the signal could be transmitted through interactions among exogenous hormone and the key regulators ARF6 (AUXIN RESPONSE FACTOR 6) and ARF8 (AUXIN RESPONSE FACTOR 8)^11–13^, and WOX11/12 (WUSCHEL-RELATED HOMEOBOX 11 and 12) which were up-regulated by the hormone from competent cells to root founder cells^14^. However, considering tea plant regeneration system, most studies mainly focused on optimization of the culture formulae and conditions, whilst few have been carried out to elucidate the regulatory effect of auxins and CKs on the dedifferentiation and redifferentiation of tea plant at molecular level.

Transcriptome analysis can reveal the presence and quantity of mRNA in a biological sample at a given moment and provide the profile of gene expression under a certain condition through ribonucleic acid sequencing (RNA-Seq) technology, providing an important way for mapping metabolic pathways and distinguishing functional genes^15^. This technology has the advantages of high throughput, low cost, high sensitivity and wide application to the species with unknown genomic sequence. Transcriptome analysis has been applied in tea plant for revealing key genes involved in response against stress, such as drought^16^, cold^17^, aluminum toxicity^18^ and disease^19^, as well as mapping the metabolic pathways including theanine biosynthesis^20^, ascorbic acid biosynthesis^21^ and nitrogen assimilation^22^. In the present study, difference of gene expression pattern in the induced primary calli, calli as well as redifferentiated roots was investigated during tissue culture of tea plant, and the cell division and redifferentiation-related regulating pathways triggered by auxins and CKs were also discussed.

## Results

### Morphological change during phase transition

**Figure 1:**
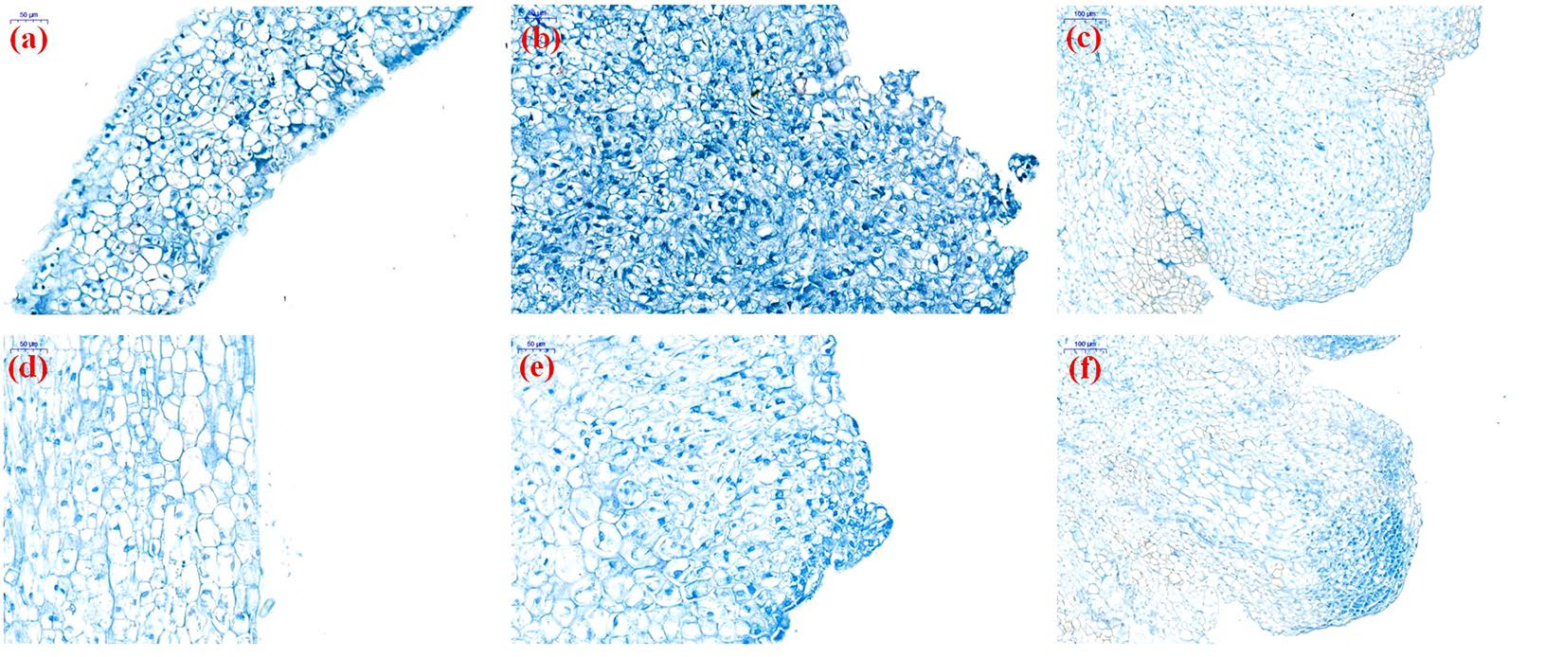
Microscopic observation of leaf- and stem- derived tissue culture samples. (**a**) stem-derived primary callus, (**b**) stem-derived callus, and (**c**) longitudinal section of regenerated root from stem-derived callus; (**d**) leaf-derived primary callus, (**e**) leaf-derived callus, and (**f**) longitudinal section of regenerated root from leaf-derived callus.

When the stem and leaf were incubated on the callus inducing medium, the cells around the wound began to become competent; many white tiny cell clusters, the primary calli, could be seen through naked eye after incubation for 10–12 days, and rapidly divided cells with relative big nucleus could be easily observed through optical microscope at this stage (Fig. 1a,d). One week later, massive cell clusters appeared around explant^23,24^ and calli formed (Fig. 1b,e). After the callus was incubated on root induced medium for more than 15 days, the root primordium began to appear, and the vascular connection was established between callus and the root primordium (Fig. 1c,f)^25–27^.

### *De novo* assembly and functional annotation

Transcripts of the explants (leaf and stem), primary calli, calli and redifferentiated roots were sequenced on Illumina Hiseq 2500 platform and average of the obtained clean data for each sample exceeded 2GB. A total of 93,607 unigenes was *de novo* assembled from the transcription data of these samples, with 784 bp in average length and 1,018 bp in N50 length; and 19,380 unigenes had a length of above 1000 bp, accounting for 20.60% of the total sequence number (Table 1). All the unigenes were compared with the reference sequences in the Nr, Swiss-Prot, KEGG, COG, KOG, GO and Pfam databases. 51,190 unigenes (54.7%) did not show significant similarity with known genes, while 42,417 unigenes (45.3%) were annotated at least in one database, of which 16,120 sequences were above 1000 bp in length (Table 2).

**Table 1:** The obtained unigene library of *C*. *sinensis* cultivar Jinxuan.

Length range	Total number	Percentage (%)
0–300	—	—
300–500	47,544	50.79
500–1000	26,783	28.61
1000–2000	12,844	13.72
2000+	6,436	6.88
Total number	93,607	/
Total length	73,398,653	/
N50 length	1,018	/
Mean length	784	/

**Table 2:** Summary of the unigene annotation in different databases.

Database	Annotated number	300 ≤ length < 1000	length ≥ 1000
COG	11,881	5,944	5,937
GO	23,477	13,938	9,539
KEGG	15,076	9,252	5,824
KOG	25,132	15,300	9,832
Pfam	28,279	14,668	13,611
Swissprot	25,852	14,578	11,274
Nr	40,131	24,215	15,916
All	42,417	26,297	16,120

### Differentially expressed genes during phase transition

**Figure 2:**
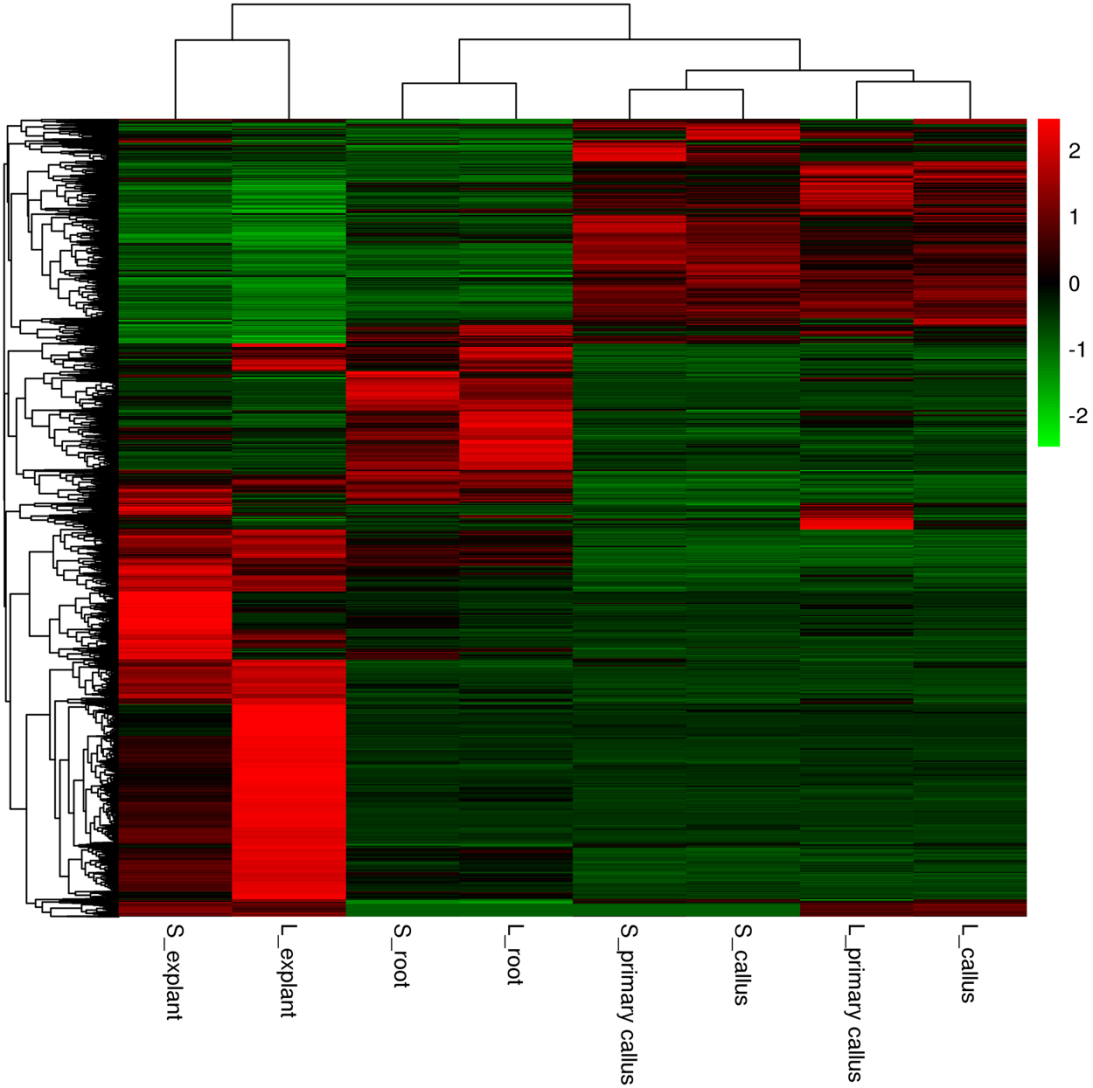
The heat-map of 7,193 DEGs shared in the 8 samples, based on Z-score normalized FPKM values in eight internode segments. S and L represented the stem and leaf. The callus was induced from the stem and leaf on the MS medium supplemented with 2,4-D (2.0 mg/L) and BAP (0.4 mg/L), the root was regenerated from the stem and leaf derived callus on 1/2 MS medium supplemented with NAA (1.5 mg/L).

**Figure 3:**
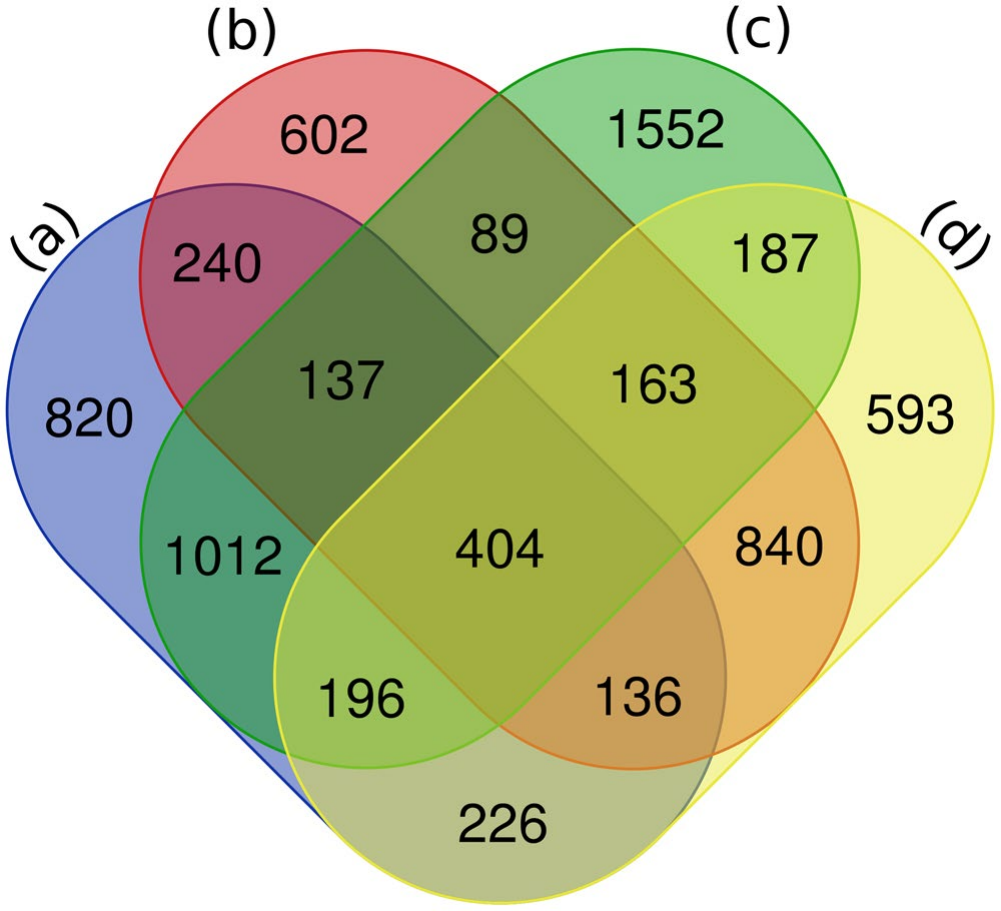
Venn diagram showed the number of DEGs observed between different samples. (**a**) DEGs between the stem explant and the callus, (**b**) DEGs between the stem derived callus and the regenerated root, (**c**) DEGs between the leaf explant and the callus, (**d**) DEGs between leaf derived callus and the regenerated root.

Analysis showed that 7,193 differentially expressed genes (DEGs) were obtained after comparison of the expression level between these samples according to the threshold of *q* value < 0.005 & |log_2_ (fold change)| > 1. Although the calli were derived from different explants, these tissues were clustered into a branch according to the gene expression behavior; similarly, the redifferentiated roots derived from stem and leaf were also clustered together (Fig. 2). More than 3,000 DEGs were observed during the phase change from stem explant to stem-derived primary callus (3,014 DEGs) and from leaf explant to leaf-derived primary callus (3,495 DEGs), respectively (Fig. 3). Among them, much more genes were down-regulated (2,133 down *vs*. 881 up in stem-derived tissues and 2,574 down *vs*. 921 up in leaf-derived tissues) during these phase changes. Similarly, a large number of DEGs were also found during the phase transition from stem-derived callus to its regenerated root (2,610) and from leaf-derived callus to its regenerated root (2,744), however, more genes were up-regulated (723 down *vs*. 1,887 up in stem-derived tissues and 937 down *vs*. 1,807 up in leaf-derived tissues) during root redifferentiation. Meanwhile, relatively fewer DEGs were found during the growth stage from stem-derived primary callus to stem-derived callus (100 down *vs*. 101 up) and from leaf-derived primary callus to leaf-derived callus (227 down *vs*. 200 up). It was clear that much more genes were required to change their expression patterns during the phase transition such as dedifferentiation and redifferentiation, while fewer genes changed their expressions at various growth periods. Similar change trends of up- and down-regulated DEGs were obtained from different initial explants (stem and leaf) during dedifferentiation and redifferentiation, indicating that the various phases or statuses of tissue culture could be determined by a set of DEGs and epigenetic variation of these genes might profoundly impact on the fate of the regenerated cells. Interestingly, the number of the down- and up-regulated genes changed reversely during dedifferentiation and redifferentiation, indicating differentiated cells required to activate much more genes for exerting special function of the cells.

### KOG classification of the DEGs

**Figure 4:**
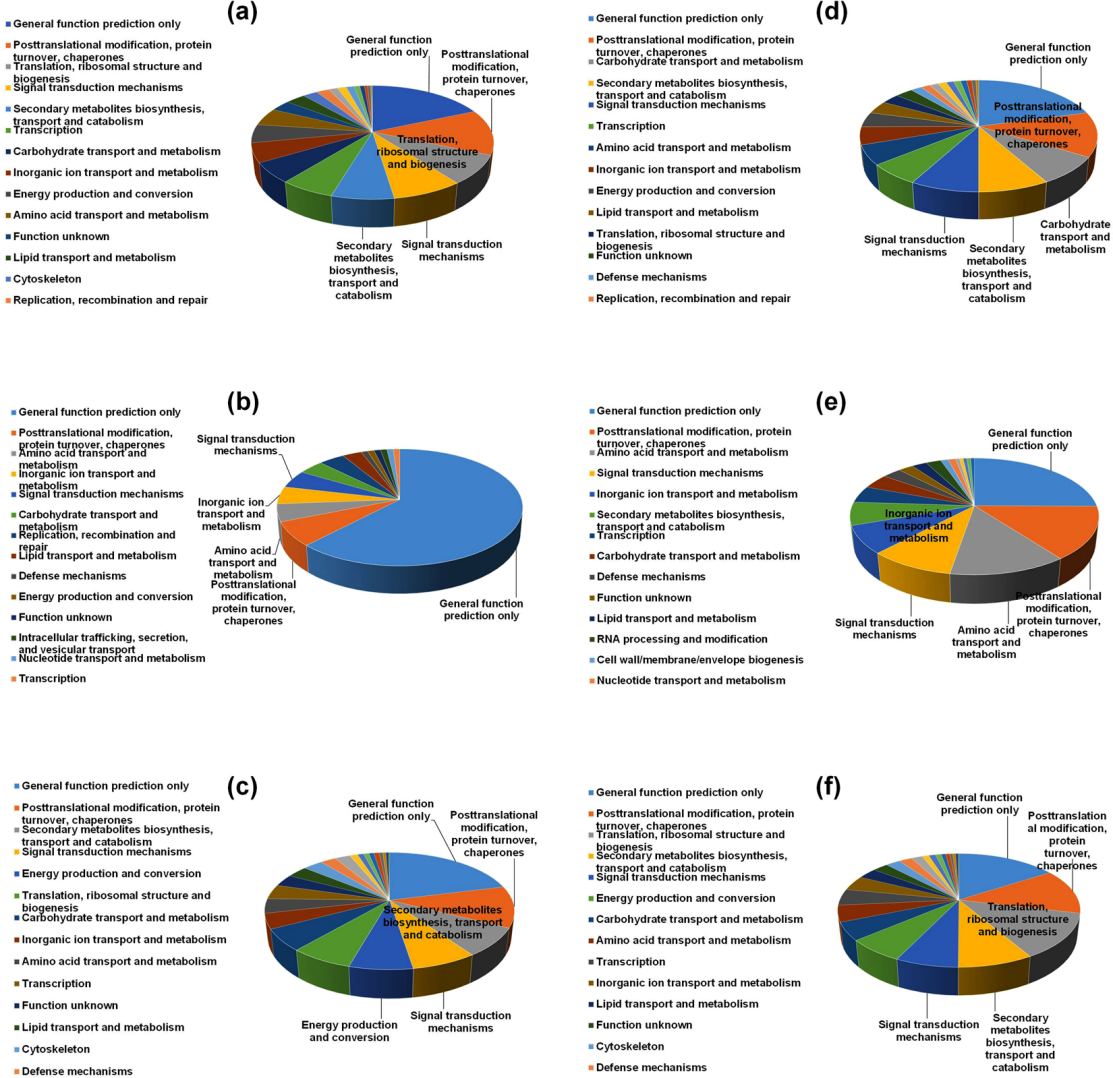
KOG annotation of the DEGs. (**a**) stem explant *vs*. stem-derived primary callus, (**b**) stem-derived primary callus *vs*. stem-derived callus, and (**c**) stem-derived callus *vs*. regenerated root; (**d**) leaf explants *vs*. leaf-derived primary callus, (**e**) leaf-derived primary callus *vs*. leaf-derived callus, and (**f**) leaf-derived callus *vs*. regenerated root.

KOG analysis showed that around 80% DEGs were mainly categorized into the classes of the “general function prediction only”, “posttranslational modification, protein turnover, chaperones”, “signal transduction mechanisms”, “secondary metabolites biosynthesis, transport and catabolism”, “carbohydrate transport and metabolism”, “transcription”, “inorganic ion transport and metabolism”, “energy production and conversion”, “amino acid transport and metabolism”, and “translation, ribosomal structure and biogenesis”, although number of the DEGs among the samples with various tissue culture status was different in these classes (Fig. 4), indicating that the expression change of the genes associated with these classes might be very important for phase transition during tissue culture since levels in the secondary metabolites, proteins, carbohydrates, inorganic ions, energetic metabolism and amino acids are likely quite different between differentiated tissues (such as leaf, stem and root) and dedifferentiated ones.

### KEGG pathway enrichment of the DEGs

**Figure 5:**
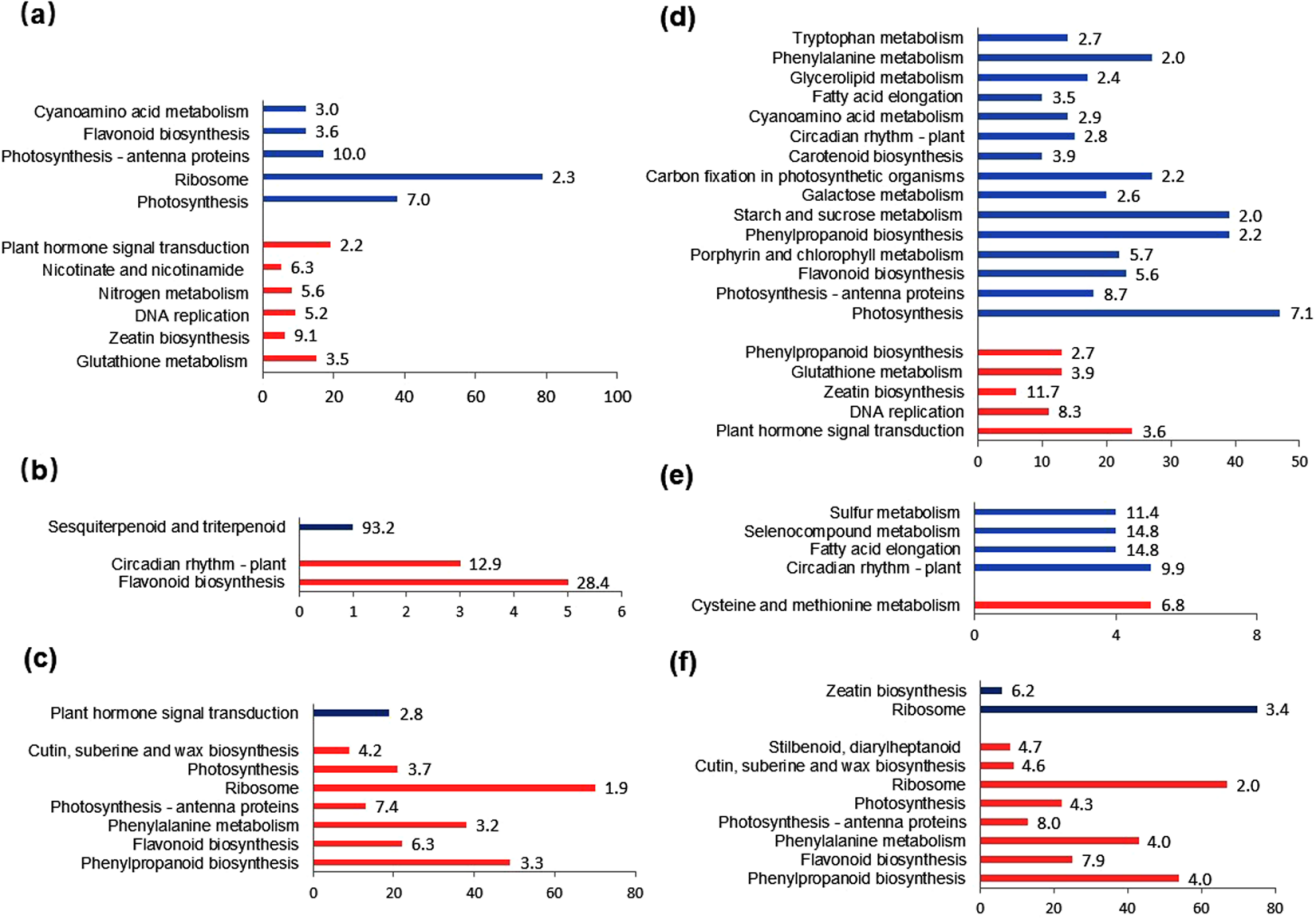
KEGG pathway analysis of the DEGs. (**a**) Stem explant *vs*. stem-derived primary callus, (**b**) stem-derived primary callus *vs*. stem-derived callus, and (**c**) stem-derived callus *vs*. regenerated root; (**d**) leaf explants *vs*. leaf-derived primary callus, (**e**) leaf-derived primary callus *vs*. leaf-derived callus, and (**f**) leaf-derived callus *vs*. regenerated root. Blue and red bar indicated the enriched gene number of the KEGG pathway in down- and up-regulated DEGs respectively; the number in front of the bar indicated the enrich factor of the KEGG pathway in down- and up-regulated DEGs (q < 0.1).

When the primary callus was formed from the stem explant, the up-regulated DEGs significantly enriched in the KEGG item of “glutathione metabolism”, “zeatin biosynthesis”, “DNA replication”, “nitrogen metabolism”, “nicotinate and nicotinamide metabolism”, and “plant hormone signal transduction”; while the down-regulated DEGs did in the item of “photosynthesis”, “ribosome”, “photosynthesis-antenna proteins”, “flavonoid biosynthesis”, and “cyanoamino acid metabolism” (Fig. 5). When the root was regenerated from the stem-derived callus, the enriched items of the up-regulated DEGs were “phenylpropanoid biosynthesis”, “flavonoid biosynthesis”, “phenylalanine metabolism”, “photosynthesis-antenna proteins”, “ribosome”, “photosynthesis”, as well as “cutin, suberine and wax biosynthesis”; the significantly enriched item of the down-regulated DEGs was “plant hormone signal transduction”. During the phase transition from leaf explant to primary callus, the pathway of “plant hormone signal transduction”, “DNA replication”, “zeatin biosynthesis”, and “glutathione metabolism” was significantly up-regulated, while pathway of “photosynthesis”, “photosynthesis-antenna proteins”, “flavonoid biosynthesis”, “porphyrin and chlorophyll metabolism”, “starch and sucrose metabolism”, “galactose metabolism”, and “carbon fixation in photosynthetic organisms” was significantly down-regulated, besides the remarkable change of the “phenylpropanoid biosynthesis”. Compared with the leaf-derived callus, the pathway of “phenylpropanoid biosynthesis”, “flavonoid biosynthesis”, “phenylalanine metabolism”, “photosynthesis - antenna proteins”, “photosynthesis”, “cutin, suberine and wax biosynthesis”, as well as “stilbenoid, diarylheptanoid and gingerol biosynthesis” was up-regulated in the regenerated root, while the pathway of “zeatin biosynthesis” was down-regulated significantly, besides remarkable change of the pathway of “ribosome”. It was apparent that up-regulation of “plant hormone signal transduction”, “zeatin biosynthesis”, “DNA replication” and “glutathione metabolism” pathways, as well as down-regulation of the photosynthesis- and secondary metabolism- related pathways were necessary for dedifferentiation of the explants although change of pathways might also be influenced by the various initial explants^28–32^. Meanwhile, root redifferentitation requires the slowdown of cell division through deactivating the signal transduction pathway, and activating the pathway related to biosynthesis of the cutin, suberine, wax, and phenylpropanoid^33^. Plastid might also be rebuilt in the rededifferentiated cell through up-regulating the photosynthesis-related pathways.

### Expression verification of auxins and CKs regulation-related genes

**Figure 6:**
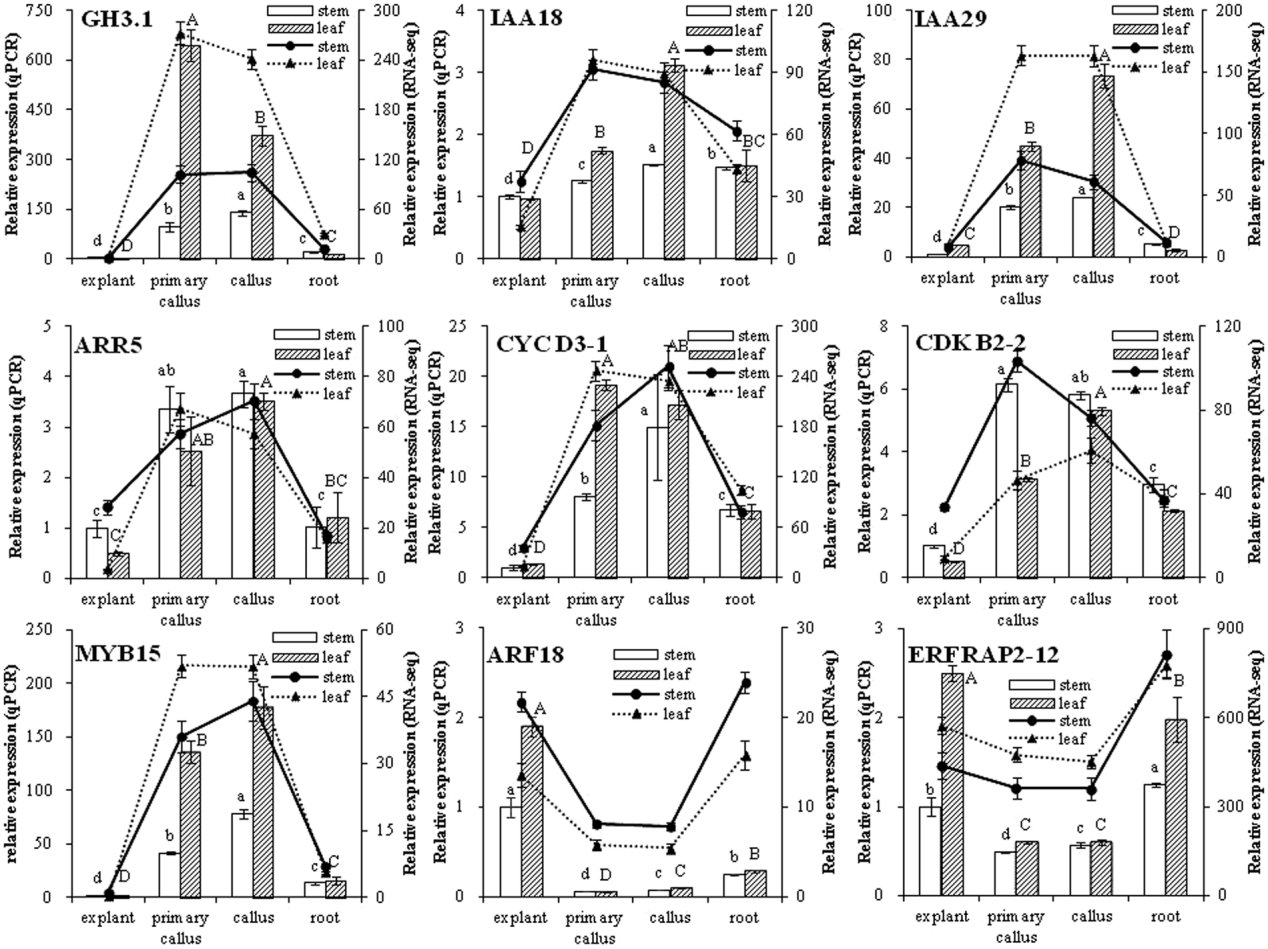
Expression validation of the auxins and cytokinins regulation -related genes by qPCR. Relative expression levels obtained from qPCR and RNA-Seq were shown as column and line respectively, and different letters (lower case for stem-derived samples, upper case for leaf-derived samples) indicated the significant difference at p < 0.05.

**Figure 7:**
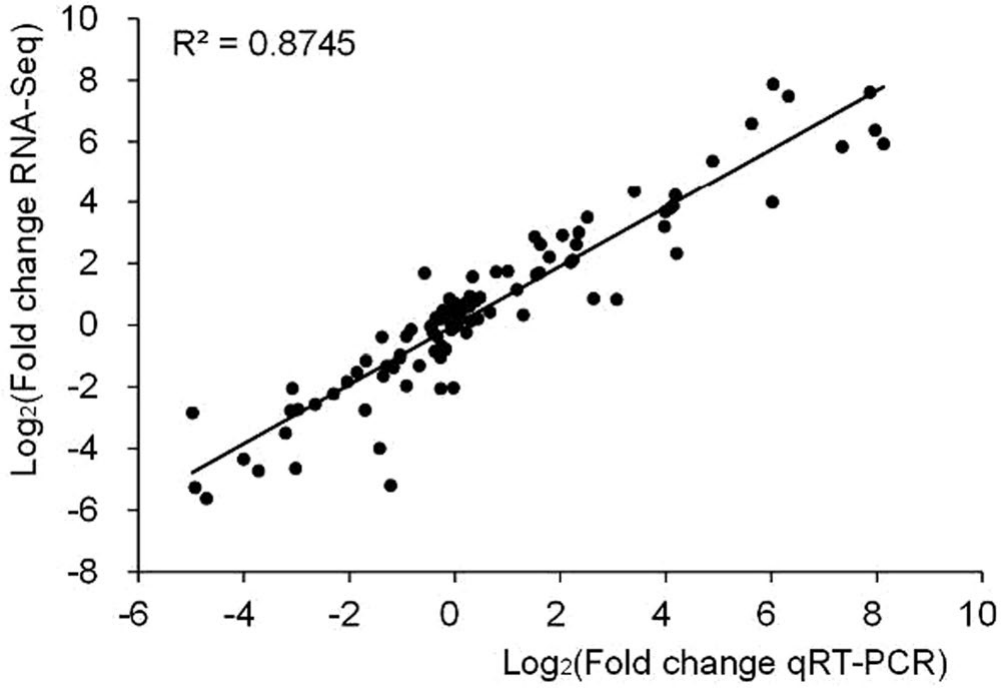
Correlation of the gene expression obtained from qPCR and RNA-seq.

As the pathway related with plant hormone signal transduction was significantly fluctuated during dedifferentiation and redifferentiation, nine DEGs, being considered to be closely related to regulation of the auxins and CKs during phase transition, were screened out (Table 3), namely indole-3-acetic acid-amido synthetase GH3.1 (*GH3*.*1*, GenBank accession No. MH051721), auxin-responsive protein IAA18 –like (*IAA18*, GenBank accession No. MH051728), auxin-responsive protein IAA29 (*IAA29*, GenBank accession No. MH051727) and auxin-responsive factor 18-like (*ARF18*, GenBank accession No. MH051722); two-component response regulator ARR5-like (*ARR5*, GenBank accession No. MH051725); transcription factor MYB15 (*MYB15*, GenBank accession No. MH051730), ethylene-responsive transcription factor ERF RAP2–12 (*ERF RAP2-12*, GenBank accession No. MH051724), Cyclin D3-1 (*CYCD3-1*, GenBank accession No. MH051723) and cyclin-dependent kinase B2-2 (*CDKB2-2*, GenBank accession No. MH051726). The expression pattern of these genes during phase transition was verified by qPCR (Fig. 6). The result showed that expression profiles of these 9 genes in the dedifferentiated and redifferentiated tissues derived from initial stem and leaf explants were quite similar to those obtained from the transcriptomic analysis, with a correlation coefficient being 0.8745 (p < 0.01) (Fig. 7). Among these 9 genes, *ARR5*, *GH3*.*1*, *IAA18*, *IAA29*, *CYCD3-1*, *CDKB2-2* and *MYB15* were up-regulated in primary callus and callus then down-regulated in the regenerated root, while the expressions of *ARF18* and *ERF RAP2-12* were down-regulated in primary callus and callus but up-regulated in the regenerated roots. These validations indicated that transcriptome analysis faithfully revealed the gene modulation mechanism during the phase changes.

**Table 3:** Auxin and cytokinins regulation-related DEGs for qPCR validation.

Gene	GenBank accession No.	Annotation	Primer pair
*GH3*.*1*	MH051721	Indole-3-acetic acid-amido synthetase GH3.1	GH3.1 F 5′-GGTATACCGACAAGGCCGAG
			GH3.1 R 5′-GCTCCTCAAAGGTCCCACTC
*ARF18*	MH051722	Auxin-responsive factor 18-like	ARF18F 5′-ACCATTCTGGAGAACCGCTG
			ARF18R 5′-ACCATTCTGGAGAACCGCTG
*CYCD3-1*	MH051723	Cyclin D3-1	CYC-D3-1F 5′-CAAGTCACGGGTCGGTAGAG
			CYC-D3-1R 5′-GTTGTACCCGAGTGTCCTCC
*RAP2-12*	MH051724	Ethylene-responsive transcription factor ERF RAP2-12	ERF RAP2-12F 5′-TGACTCGGACACACTCTCCT
			ERF RAP2-12R 5′-TTTGGGGGTGAGACCTTTGG
*ARR5*	MH051725	Two-component response regulator ARR5-like	ARR5F 5′-GGCATCGGAAAAACGGGTTG
			ARR5R 5′-TCAGTGCCATCAGACGAAGG
*CDKB2-2*	MH051726	Cyclin-dependent kinase B2-2	CDKsF 5′-TGCACTTCCAACGCACAATC
			CDKsR 5′-GCCTCATCAAAACCTCGCTTC
*IAA29*	MH051727	Auxin-responsive protein IAA29	IAA29F 5′-TGCACTTCCAACGCACAATC
			IAA29R 5′-GCCTCATCAAAACCTCGCTTC
*IAA18*	MH051728	Auxin-responsive protein IAA18	IAA18F 5′-CCCCAACAAGCATCCTGTCT
			IAA18R 5′-ATCCAGAACAAGCACGAGGG
*MYB15*	MH051730	Transcription factor MYB15	MYB15F 5′-CTCCTCTTCATTGGCAGGTCC
			MYB15F 5′-CGAACCGACAACGAGATCAA

## Discussion

**Figure 8:**
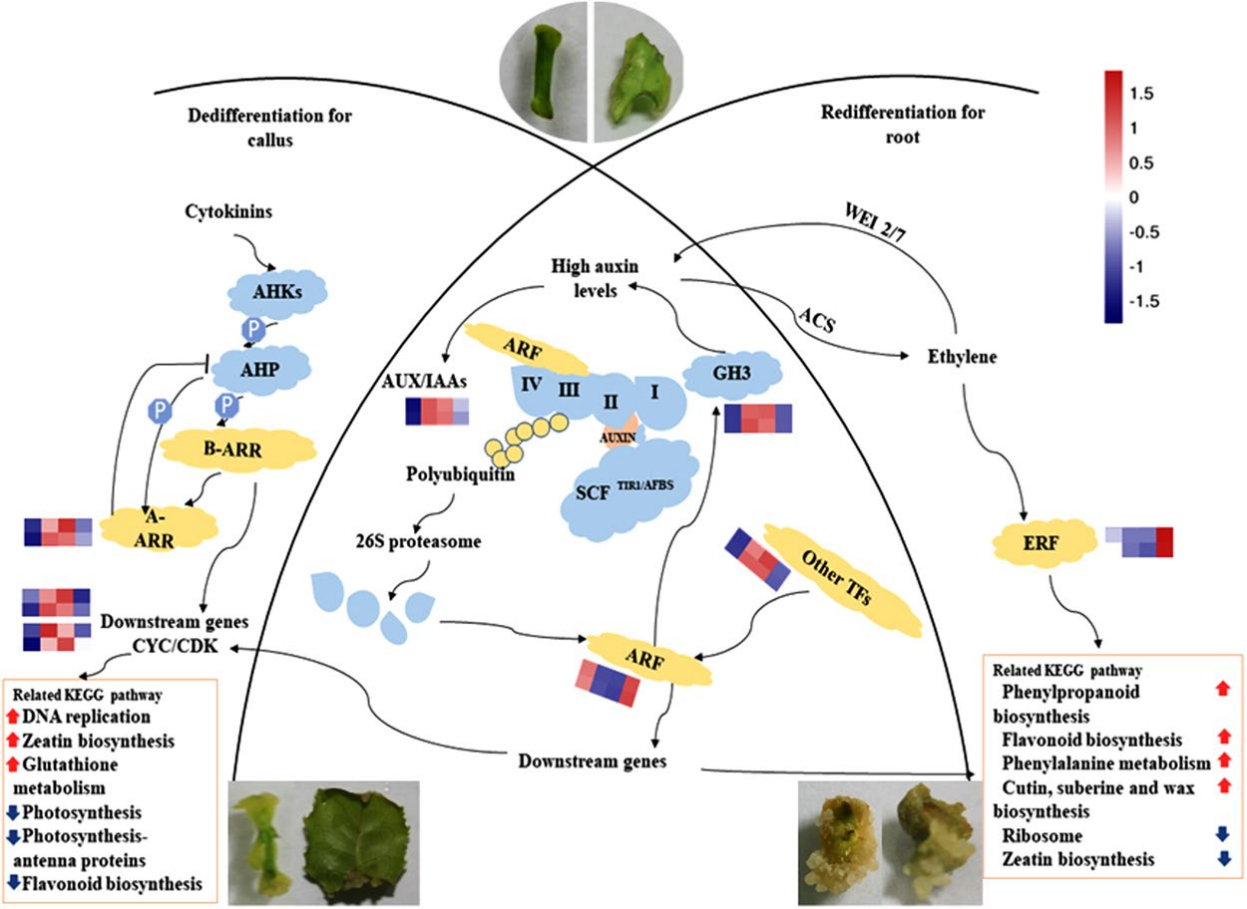
The proposed hormone-related gene modulation during dedifferentiation and redifferentiation of tea plant. AHKs: Arabidopsis histidine kinases; AHPs: Arabidopsis histidine-containing phosphotransfer proteins; A-ARR: A-type Arabidopsis response regulator; B-ARR: B-type Arabidopsis response regulator; CYC/CDK: cyclin/yclin-dependent kinase; AUX/IAAs: auxin/indole-3-acetic acid family proteins; SCF: S-phase kinase-associated protein- cullin-F box; TIR1/AFB: transport inhibitor response 1/auxin-related F-box; ARF: auxin responsive factor; GH3: Gretchen Hagen 3; Other TFs: transcription factors including MYB15; WEI2/7: weak ethylene insensitive 2/7; ACS: 1-aminocyclopropane- 1-carboxylate synthase; ERF: ethylene-responsive transcription factor. Expression levels in stem/leaf-derived tissues of tea plant obtained from RNA-seq were indicated as color bar and normalized by z-score processing.

Exogenous plant growth regulators are usually applied to induce specific cell types and/or organs during dedifferentiation and redifferentiation of tissue culture. In this study, 2,4-dichlorophenoxyacetic acid (2,4-D, belonging to auxins) and 6-benzylaminopurine (BAP, belonging to CKs) were used for inducing callus from stems and leaves of tea plant. The transcriptome and qPCR results revealed that many DEGs, especially the hormone-related genes, were significantly triggered by the growth regulators treatment, and finally initiated the dedifferentiation process. As shown in Fig. 8, the cell division and callus formation might be induced and facilitated through combination of the two regulating pathways. One was auxin-related pathway, in which the expression of auxin-responsive protein encoding gene *IAA18* and *IAA29* were up-regulated by addition of exogenous 2,4-D, the increased gene products of the *IAA18* and *IAA29* would then accelerate the release of the ARFs through combination with different TIR1/AFB (transport inhibitor response 1/auxin-related F-box protein) and ARF proteins and activating the ubiquitinoylation, then the excited ARFs would up-regulate the expression of the downstream gene *CDK B2-2*^34–36^. The previous reports concerning early gene response to auxin treatment also showed that Aux/IAA are short-lived nuclear proteins and mostly have four conserved domains, where the domain III and IV are responsible for dimerization with other Aux/IAA or heterodimerization with auxin responsive factors (ARFs), and the domain II contains the degron motif and can interact directly with the TIR1/AFB and auxin, leading to the ubiquitination and degradation *via* 26 S proteasome at higher auxin levels and consequently releasing ARFs to regulate the expression of downstream genes^37,38^. Another was CK-related pathway, in which the expression of response regulator gene *B-ARR*s (B-type Arabidopsis response regulators) and *A-ARR5* were all phosphorylated through AHKs (Arabidopsis histidine kinases) → AHPs (Arabidopsis histidine-containing phosphotransfer proteins) phosphorelay signaling way^39^, and cyclin gene *CYC D3-1* was up-regulated as the downstream gene of the phosphorylated *B-ARR*s, due to the addition of BAP. Our result confirmed that dedifferentiation process is also closely related to the CKs modulation pathway which has been reported in *Arabidopsis*. When the cell is contacted with CKs, transmembrane receptors, AHKs will transmit signals to the nucleus *via* the phosphorelay pathway, leading to phosphorylation and activation of transcription factors B-ARRs^40^, B-type ARRs then sequentially induce the expression of some response genes, including *A-ARRs*^41^ and *cyclin* (*CYC*)^42^. In addition, extremely up-regulated transcription factor *MYB15* was also observed, indicating this gene might also be involved in modulation of the dedifferentiation because MYB15 could modulate the expression of auxin-inducible genes by interacting with ARFs^43^ and affect the plant growth and development^34^. Our study also revealed that a GH3 family protein encoding gene *GH3*.*1* was significantly up-regulated in callus, which was consistent with other previous finding that GH3 can maintain auxin homeostasis by conjugating excessive indole-3-acetic acid to amino acids in the presence of a higher level auxin^44^. The up-regulated expression of the *MYB15*, *IAA18* and *IAA29* could promote auxin signaling pathways through interactions with related ARFs; up-regulating *CDK B2-2* and *CYC D3-1*, involved in auxin and CKs signaling pathways, might be necessary for callus induction of tea plant. The up-regulation of *GH3*.*1* and *A-ARR5* might be a response of tissues against high hormonal levels. After the genes related to signaling transduction changed their expression, many downstream pathways including DNA replication, zeatin biosynthesis, glutathione metabolism and photosynthesis were also be modulated. Zeatin biosynthesis and DNA replication were modified for initiating cell division to form callus^28–30^, while photosynthesis was changed due to dedifferentiation of chloroplast. Initiation and proliferation of the callus require the up-regulation of glutathione metabolism to deal with the antioxidant defense, nutrient requisition, and regulation of cellular events^31,32^.

When the root was regenerated from the callus in the 1/2 MS medium supplemented with 1.5 mg/L naphthalene acetic acid (NAA), the expression level of *IAA18* and *IAA29* were quite lower than that in callus stage, but similar to that in initial explants; while the genes encoding the auxin-responsive factor ARF18 and ethylene-responsive transcription factor ERF RAP2-12 were remarkably up-regulated compared with the callus stage. According to the published reports, auxin application could promote expression of the *ACS4* gene encoding 1-aminocyclopropane-1-carboxylate (ACC) synthase to induce ethylene biosynthesis^45^, leading to enhanced ethylene level and up-regulating expression of ethylene-responsive transcription factor. Furthermore, ethylene could influence auxin level and further facilitate auxin signaling pathway^46^ through modulating the expression of the weak ethylene insensitive 2 and 7 (*WEI2* and *WEI7*) which encoded the rate-limiting enzyme anthranilate synthase in the tryptophan biosynthesis^47^. Thus, up-regulating expression of *ARF18* and *ERF RAP2-12*, and down-regulating expression of *IAA18* and *IAA29* might be essential for root induction, as well as changes in gene expression of some downstream pathways, such as cutin, suberine, wax, and phenylpropanoid metabolism which is required to form the specialized cell wall through activating the pathway related to biosynthesis of the cutin, suberine, wax, and phenylpropanoid^33^.

## Methods and Materials

### Preparation of tea plant samples

Seedlings of *Camellia sinensis* cultivar ‘Jinxuan’ derived through *in-vitro* technique were micropropagated and maintained on the Murashige and Skoog (MS) medium with addition of 2 mg/L 6-benzylaminopurine (BAP), 0.1 mg/L naphthalene acetic acid (NAA), 30 g/L sucrose and 9 g/L agar through single-node culture. A callus inducing medium (CIM) was prepared and autoclaved at 121 °C for 20 min after adding 2.0 mg/L 2,4-dichlorophenoxyacetic acid (2,4-D), 0.4 mg/L BAP, 30 g/L sucrose and 9 g/L agar into MS medium, and a root inducing medium (RIM) was also prepared and autoclaved after adding 1.5 mg/L NAA, 30 g/L sucrose and 9 g/L agar into 1/2 MS medium. pH value of the all the media was adjusted to pH5.8.

The stems and leaves were disassociated from the seedlings and cut into small pieces (~0.5 cm for stem and ~0.5 cm^2^ for leaf) and used as explants. The explants were inoculated onto the CIM and cultivated in tissue culture room at 24 ± 1 °C under the dark condition. The callus began to appear from the cuts of the stem explant (S-explant) after 10 days, and then surrounded the explant after 16 days. Sampling was conducted on the 10^th^ day and 16^th^ day, designated as S-primary callus and S-callus, respectively. The stem-derived callus was then inoculated onto the RIM, the root was induced after 15 days. Sampling was carried out and designated as S-root. Similarly, the leaf-derived primary callus and callus (L-primary callus and L-callus) were also obtained after cultivating the leaf explant (L-explant) on the CIM for 12 and 20 days, and redifferentiated root (L-root) was sampled after inducing the L-callus on the RIM for 20 days. The obtained samples were immediately frozen in liquid nitrogen and stored at −80 °C for further use. All the tests were conducted in biological triplicates.

### Observation of the callus and regenerated root

In order to confirm the phase transition of the tissue culture, samples were embedded in paraffin and stained with aniline blue solution^48,49^. Observation was performed on a Nikon Eclipse E100 (Nikon Co., Ltd, Japan) with a Nikon DS-U3 image capture.

### RNA extraction

RNA extraction was carried out with RNAprep Pure Plant Kit (Tiangen Biotech Co., Ltd., Beijing, China) according to the manufacturer’s instruction. The purity of RNA was checked by NanoPhotometer® spectrophotometer (Implen, CA, USA). The concentration of RNA was measured on Qubit® 2.0 Flurometer (Life Technologies, CA, USA) by using Qubit® RNA Assay Kit. The integrity of RNA was assessed on Bioanalyzer 2100 system (Agilent Technologies, CA, USA) by using RNA Nano6000 Assay Kit.

### Transcriptome analysis

The mRNAs were enriched by using Oligo (dT) magnetic beads from the 3 μg total RNA for each sample. Sequencing libraries were generated using NEBNext®Ultra™ RNA Library Prep Kit for Illumina® (NEB, USA) according to the manufacturer’s recommendations and index codes were added to attribute sequences to each sample. The clustering of the index-coded samples was performed on a cBot Cluster Generation System using TruSeq PE Cluster Kit v3-cBot-HS (Illumia, NEB, USA) according to the manufacturer’s instructions. After cluster generation, the libraries were sequenced on an Illumina Hiseq 2500 platform and paired-end reads were generated. Clean data were obtained by removing reads containing adapter, reads containing ploy-N and low quality reads from raw data. Transcriptome assembly was accomplished from the clean data using Trinity software. Gene function of all non-redundant transcripts was annotated based on the database of the NCBI nonredundant protein sequences (Nr), Protein family (Pfam), Eukaryotic Orthologous Group/Clusters of Orthologous Groups of proteins (KOG/COG), a manually annotated and reviewed protein sequences (Swiss-Prot), Kyoto Encyclopedia of Genes and Genomes (KEGG) and Gene Ontology (GO) by using BLASTALL package with the significant threshold of E-value ≤ 10^−5^. FPKM was used as expression strength of the gene, and differential expression analysis of two samples was performed using the DEGseq R package (ver. 2.1.0). *p* value was adjusted using *q* value and *q* value < 0.005 & |log2 (fold change)| > 1 was set as the threshold for significantly differential expression. Samples of two biological replicates at each culture stage were used for transcriptome analysis.

### Quantitative real-time PCR analysis

To validate the gene expression results of transcriptome, qRT-PCR was employed to determine the expression of 9 genes related to auxins and cytokinins regulation (Table 3) on Applied Biosystems™ StepOnePlus™ Real-Time PCR System (ABI, Carlsbad, CA, USA) by using SYBR Premix Ex TaqTM II (TaKaRa Biotechnology Co., Ltd., Dalian, China) according to the previous paper^50^. PCR primers were designed by primer-blast (https://www.ncbi.nlm.nih.gov/tools/primer-blast/) with default parameters except the product size (100–250 bp), and the primers were ordered from Sangon Biotech (Shanghai) Co., Ltd. The PCR cycling conditions were: 40 cycles of 95 °C for 30 s, 55 °C for 5 s and 60 °C for 30 s. The melting-curves were analyzed during the reactions to ensure reaction specificity. The relative expression levels of the different genes were calculated by the 2^−ΔΔCt^ method^51^ by using β-actin gene as control. qPCR analysis was performed in three biological replicates with 3 technical replicates for each biological replicate.

## Data Availability

The datasets generated during and/or analysed during the current study are available from the correspondingauthor on reasonable request.
